# Impact of an integrated mother-preterm infant intervention on birth hospitalization charges

**DOI:** 10.1038/s41372-019-0567-7

**Published:** 2020-01-08

**Authors:** Susan C. Vonderheid, Chang G. Park, Kristin Rankin, Kathleen F. Norr, Rosemary White-Traut

**Affiliations:** 1 Department of Women Children, and Family Health Science, College of Nursing, University of Illinois at Chicago, Chicago, IL USA; 2 College of Nursing, University of Illinois at Chicago, Chicago, IL USA; 3 Division of Epidemiology and Biostatistics, School of Public Health, University of Illinois at Chicago, Chicago, IL USA; 4 Children’s Hospital of Wisconsin, Milwaukee, WI USA

**Keywords:** Health care economics, Health services, Health care economics, Health services

## Abstract

**Objective:**

To examine whether the H-HOPE (Hospital to Home: Optimizing the Preterm Infant’s Environment) intervention reduced birth hospitalization charges yielding net savings after adjusting for intervention costs.

**Study design:**

One hundred and twenty-one mother-preterm infant dyads randomized to H-HOPE or a control group had birth hospitalization data. Neonatal intensive care unit costs were based on billing charges. Linear regression, propensity scoring and regression analyses were used to describe charge differences.

**Results:**

Mean H-HOPE charges were $10,185 lower than controls (*p* = 0.012). Propensity score matching showed the largest savings of $14,656 (*p* *=* 0.003) for H-HOPE infants, and quantile regression showed a savings of $13,222 at the 75th percentile (*p* = 0.015) for H-HOPE infants. Cost savings increased as hospital charges increased. The mean intervention cost was $680 per infant.

**Conclusions:**

Lower birth hospitalization charges and the net cost savings of H-HOPE infants support implementation of H-HOPE as the standard of care for preterm infants.

## Introduction

More than half a million infants are born prematurely (<37 weeks gestation) in the United States annually [1]. The biologic risk of prematurity places infants at greater risk for suboptimal growth and development, poor behavioral organization, and chronic and acute morbidities than full-term infants. These adverse outcomes result in substantially greater healthcare utilization and expenditures during the birth hospitalization, childhood and into adulthood [2–6]. Employer-sponsored health plans estimated expenditures of $14 billion for preterm infants during the first year of life in 2015 [7]. Although comprising 9.1% of all births, preterm or low birth weight infants accounted for 43.4% of the total costs of birth hospitalizations in the 2009 Healthcare Cost and Utilization Project [8]. This study also showed that mean costs of birth hospitalization for preterm or low birth weight infants ranged from $8,000 for uninsured/self-pay infants, $15,300 for infants covered by commercial payers, and $16,200 for infants covered by Medicaid [8]. A review of ten studies found that despite study date, location and methodology, birth hospitalization costs among moderate or late preterm infants (32–37 weeks) were at least twice as high compared to full-term infants [9]. Among preterm infants 29 to 34 weeks gestation born from 2000 to 2009, mean hospitalization costs ranged from $9,740 to $52,998 [10]. Consistent with previous studies, a review of 18 studies found an inverse relationship between costs and gestational age (GA) [11].

The biologic risk of prematurity and associated alterations in mother-preterm infant interactions affect early infant health, leading to high levels of healthcare resource utilization and costs during the birth hospitalization. Premature infants’ behavioral cues are subtle and difficult for mothers, unfamiliar with premature infants, to perceive correctly and respond appropriately to infants’ behaviors [12, 13] and contributes to stress, anxiety and depression [14, 15]. The infant’s immature behavioral organization, coupled with maternal distress, place mother-premature infant dyads at risk for development of maladaptive interaction patterns [16, 17] and likely contribute to delays in feeding progression, growth and development.

While several studies have tested interventions to optimize infant development and behavior initiated during the neonatal intensive care unit (NICU) stay [18–25], we identified only one early intervention study that reported a cost analysis [26]. Hospital costs were based on adjusted charges and calculated based on estimated median treatment charges (including accommodation and ancillary) of $1250 per day in the NICU. The mean length of stay was 4 days shorter for intervention infants, resulting in lower charges of $5,000 per infant in 2001. Direct costs of the intervention (parent education and skill building) were $136 per infant, for a net savings of $4,864. The dearth of evidence about effective early interventions for programs for preterm infants and their associated costs of birth hospitalization warrants further study.

A more recent early intervention program is the Hospital to Home: Optimizing the Preterm Infant’s Environment (H-HOPE) intervention aimed at promoting early infant development and parental engagement, developed by White-Traut, Norr, and colleagues [27, 28]. Their earlier research focused on H-HOPE’s infant-directed component, the ATVV, that provides Auditory (voice), Tactile (moderate touch massage), Visual (eye to eye), and Vestibular (rocking) stimulation. The ATVV improved infant behavior, growth and development, and reduced length of hospital stay [12]. In response to mothers’ reported need for participatory guidance and social support to engage with their preterm infants, the parent-directed component of H-HOPE was developed. This component included two hospital and two home visits by a nurse-advocate team. Together, H-HOPE’s components were intended to optimize early infant behavior and parental capacity to engage in positive maternal-infant interactions [27]. A randomized controlled trial (RCT) of H-HOPE found that H-HOPE infants exhibited more alert behavioral states, improved feeding-related behaviors [29], gained weight and grew in length more rapidly [13]. Although previous research with the ATVV intervention yielded differences in length of birth hospitalization [25], the RCT of H-HOPE with infants 29–34 weeks gestation found no difference in length of stay [13]. The H-HOPE RCT also resulted in fewer acute visits between hospital discharge and 6 weeks corrected age [30].

Reducing early healthcare costs is an important potential benefit of early infant intervention given the high costs of preterm infants. This analysis examined whether the H-HOPE intervention infants had lower total charges during the birth hospitalization and yielded net savings after adjusting for the direct costs of the intervention. We expected the improved feeding, growth and development outcomes for H-HOPE infants would result in less resource use and thus, lower charges.

## Materials and methods

### Design

This cost analysis reports the direct costs of the intervention and total charges for the birth hospitalization collected during a RCT that examined the effect of H-HOPE on maternal and preterm infant outcomes.

### Sample and setting

The H-HOPE study enrolled mother-preterm dyads at two community hospitals that had either a Level II (with expanded capabilities) or a Level III NICU. These hospitals provided care to families with diverse ethnic and socioeconomic backgrounds from disadvantaged urban neighborhoods in a large midwestern city. Mothers were eligible if they were biological mothers who had at least two of the following social-environmental risk factors: self-identity as Black or Latina, less than high school education, less than 18 years of age, history of or current mental illness, family income less than 150% of the poverty line, more than one child under 24 months, 4 or more children under age 18 in household, and/or resided in a disadvantaged neighborhood. Mothers were excluded if their medical records indicated a positive screen for illicit drug use or if they had lost legal guardianship of their infants. Infants were eligible if they were born between 29 and 34 weeks GA, had no other major health problems and were clinically stable at enrollment. Infant exclusion criteria included congenital anomalies, necrotizing enterocolitis, brain injury, chronic lung disease, HIV, and prenatal drug exposure. This paper includes data for 121 mother–infant dyads enrolled in the study at discharge and for whom birth hospitalization charge data were available. Detailed information about sample sizes at enrollment, randomization using computer-generated lists and follow-up were previously reported [13]. The evaluation team was blinded to study assignment. This study was approved by the Institutional Review Board committees for the university and the two hospital sites. Informed consents were obtained from mothers soon after birth.

### Intervention and control group description

The *H-HOPE intervention* is an integrated maternal and infant intervention designed for mother-preterm infant dyads [27]. The infant-directed component is the multi-sensory intervention (ATVV, Auditory, Tactile, Visual and Vestibular) which provides 10 min of auditory (infant- directed motherese voice), tactile (moderate touch stroking or massage) and visual (eye to eye) stimulation, followed by 5 min of vestibular stimulation (horizontal rocking) [20]. The infant received the ATVV twice daily prior to feeding, beginning at 32 weeks post-menstrual age or at entry into the study for infants born at 33–34 weeks. The ATVV was provided by the mother when she was present or the in-hospital staff nurse participating in the research.

The mother-directed component of H-HOPE consisted of individualized participatory guidance regarding preterm infants by a trained nurse-community advocate team provided during 2 in-hospital visits, 2 home visits and 2 phone calls after discharge. The intervention and its fidelity are described in White-Traut et al. [12]; Burns et al. [20]; White-Traut and Norr [27].

The *attention control condition* was designed to provide a similar amount of contact with the mother and staff attention, but with distinctly different content from H-HOPE. Control group mothers received educational content that included premature infant care and car safety videos at two in-hospital sessions and four phone calls after the infant’s discharge to home regarding infant care including bathing, sleep positions and sleep habits, holding the baby, and safety of infant equipment.

### Measures

#### Infant birth hospitalization charges

Infant birth hospitalization charges were based on total charges provided by billing data from 2008 to 2011. We used charges rather than actual costs for our analysis because this was the only financial data available at both institutions. The net cost (or savings) of H-HOPE per infant was calculated by subtracting the mean direct intervention costs from the mean difference in hospital charges between study groups.

#### Costs of the intervention

Direct costs of the intervention included personnel time (average salary plus fringe benefits) and materials for training and implementing the intervention during the NICU stay. Personnel costs included the intervention trainer, the nurse-advocate team providing two in-hospital teaching visits and the in-hospital nurses providing the ATVV for the infant when the mother was not there. Average salaries and fringe benefits were obtained from human resource records. Time to attend training and time to provide each component of the intervention (the infant-directed ATVV and the participatory guidance teaching sessions for the mother) were obtained from logs recorded by nurses. Materials included a training manual for nurses and the community advocate, and educational handouts for the mother. Because this analysis focuses on birth hospitalization costs, we did not include the post-discharge costs (two home visits and two telephone calls by the nurse-advocate team). Costs for intervention development, development of outcome measures and outcome evaluations were also excluded from analysis because their costs would not be needed to implement the intervention clinically.

#### Covariates

Infant characteristics used as covariates included sex, GA at birth, birth weight, plurality (multiple birth or singleton), 5-min Apgar score, pre-discharge Problem-Oriented Perinatal Risk Assessment System score (POPRAS) [31] score and hospital length of stay. The POPRAS score was designed to predict medical risk for mortality in the perinatal and neonatal periods, with higher POPRAS scores indicating more severe neonatal morbidity [31].

Maternal baseline characteristics used as covariates included age, race/ethnicity (African–American or Latina), if interviewed in English or Spanish, education, full-time or part-time work status prior to delivery, annual household income, living situation (with baby’s father, with mother or other adult, alone), high childcare burden and living in a disadvantaged neighborhood. Education was categorized as low for women 20 or older who did not have a high school degree or GED and for women <20 who did not finish high school or were not currently still in school. Annual income was dichotomized to <185% federal poverty level (FPL) or ≥185% of the FPL. Childcare burden was defined as high if there were four or more children in the household, or another child under two years old other than the study infant (not including multiple births). Neighborhood disadvantage was derived using 5-year estimates (2005–2009) from the American Community Survey at the census tract level [32] using the Index of Neighborhood Disadvantage Score (INDS) [33]. Women’s neighborhoods were considered disadvantaged if the INDS was greater than zero.

Additional covariates for the mothers included three psychosocial variables [34, 35] measured at baseline. Depression was assessed using the Center for Epidemiologic Studies-Depression Scale (CES-D) [34]. Women who screened high on the CES-D (≥16) were classified as depressed. Maternal trait anxiety was assessed using the trait subscale of the State-Trait Anxiety Inventory (STAI) [36]. Scores range from 20–80, with a higher score indicating higher anxiety. Social support was measured using the Personal Resource Questionnaire 2000 (PRQ2000) [37, 38]. Scores were dichotomized into low (≤25th percentile) and moderate/high (>25th percentile) levels of social support. All psychosocial measures have been widely used with this population and have established validity and reliability [37].

### Data analysis

We initially examined study group equivalence for maternal and infant characteristics using Chi-square tests and *t*-tests. We also examined the distribution of hospital charges for each groups using data visualization and summary statistics. The nonparametric, kernel density estimation (KDE) is a technique that allows the investigator to create a smooth curve given a set of data [39]. This can be useful if the investigator wants to visualize the “shape” of data as a continuous representation rather than using a discrete histogram. The advantage of using KDE to compare distributions between groups is that the data speak for themselves without the arbitrariness of parametric specifications. Our next step was to model the differences in total hospital charges between study groups after adjusting for potential confounding maternal and infant characteristics. Because hospital cost data are typically highly skewed and heteroscedastic, we used three alternative estimation methods to take these distribution characteristics into account when examining the effect of the H-HOPE intervention on charges and net cost. Our first method was multiple linear regression. We then repeated the analysis using propensity score matching [40]. The third method, quantile regression, was selected to yield a robust estimation and address the limitations of log transformation [41] typically used for highly skewed data [42–44]. Log transformation can bias the estimation related to a change in the true distributional characteristic of the outcome and assumes the program effect is homogeneous across all participants [41]. The quantile regression model allowed us to examine whether the impact of H-HOPE is unequal across the distribution of charges. Results were considered significant where *p* < 0.05. Analysis was conducted using Stata statistical software [45].

To obtain the net cost difference per infant we first calculated the total direct costs of the intervention by summing personnel time and materials. Next, we divided the sum of the total direct intervention costs by the number of infants to determine the mean direct costs of the intervention per infant. Lastly, we calculated the net cost difference per infant by subtracted the mean per infant intervention costs from the mean birth hospitalization charges per H-HOPE infant estimated by linear regression.

## Results

There were no statistically significant differences by study group in infant or maternal characteristics (Table 1). For all infants in the sample, the mean unadjusted total charge was $62,408 and the median was $52,186. This difference of almost $10,000 between the mean and the median indicates a highly skewed distribution with a relatively small number of infants contributing greatly to mean charges.

**Table 1:** Infant and maternal characteristics by study condition.

	H-HOPE (*n* = 67) %, mean (SD)	Attention control (*n* = 76) %, mean (SD)	p*
Sex			
Female	37 (55.2%)	34 (44.7%)	0.21
Male	30 (44.8%)	42 (55.3%)	
Plurality			
Singleton	60 (88.2%)	67 (88.2%)	0.79
Twin/triplet	7 (10.4%)	9 (11.8%)	
Gestational age	32.22 (1.668)	32.54 (1.553)	0.24
Birth weight, g	1804.61 (373.744)	1863.26 (445.183)	0.40
Apgar score at 5 min	8.31 (0.988)	8.28 (1.078)	0.83
Infant morbidity during initial hospitalization (POPRAS)	68.95 (20.956)	71.99 (19.242)	0.37
Length of stay, days	23.15 (12.155)	22.10 (12.936)	0.62
Hospital charges $	59,385.71 (31,951.05)	68,350.20 (45,971.01)	0.22
Age, years	25.554 (6.250)	26.323 (6.651)	0.41
Race/ethnicity			
African–American	44 (47.8%)	50 (50.5%)	0.71
Latina	48 (52.2%)	49 (49.5%)	
Language preference			0.51
English	59 (64.1%)	68 (68.7%)	
Spanish	33 (35.9%)	31 (31.3%)	
Education^a^			0.91
Low for age	29 (31.9%)	32 (32.7%)	
Appropriate for age	62 (69.1%)	66 (67.3%)	
Parity			0.48
Primiparous	38 (41.3%)	36 (36.4%)	
Multiparous	54 (58.7%)	63 (63.6%)	
Income as a % of the FPL			0.78
<185%	77 (88.5%)	88 (89.9%)	
≥185%	10 (11.5%)	10 (10.2%)	
Disadvantaged neighborhood (INDS)			0.49
Yes	37 (40.2%)	35 (35.4%)	
No	55 (59.8%)	64 (64.0%)	
Employment status			0.25
Employed	40 (44.0%)	35 (35.7%)	
Not employed	51 (56.0%)	63 (64.3%)	
Living situation			0.66
With baby's father	49 (53.8%)	59 (60.2%)	
With mother or other adult only	28 (30.8%)	25 (25.5%)	
Single	14 (15.4%)	14 (14.3%)	
Depressed (CES-D)			0.31
Not depressed	63 (70.0%)	72 (76.6%)	
Depressed	27 (30.0%)	22 (23.4%)	
Trait anxiety (STAI-y2)	30.967	29.242	0.161
Social support (PRQ-2000)			0.114
Low	26 (28.57%)	18 (18.75%)	
High	65 (71.42%)	78 (81.25%)	

*SD* standard deviation; *INDS* Index of Neighborhood Disadvantage Score; *CES-D* Center for Epidemiologic Studies— Depression Scale, *FPL* federal poverty level; POPRAS Problem-Oriented Perinatal Risk Assessment System score; PRQ-2000 Personal Resources Questionnaire (2000), STAI-y2 Spielberger State-Trait Anxiety Inventory (Trait)

^a^Education is considered appropriate for age if woman is 20 or older and has a high school degree or GED, or if a women is younger and has a high school degree or is still enrolled in school

*Chi-square test for categorical and *t*-test for continuous infant characteristics

**Fig. 1:**
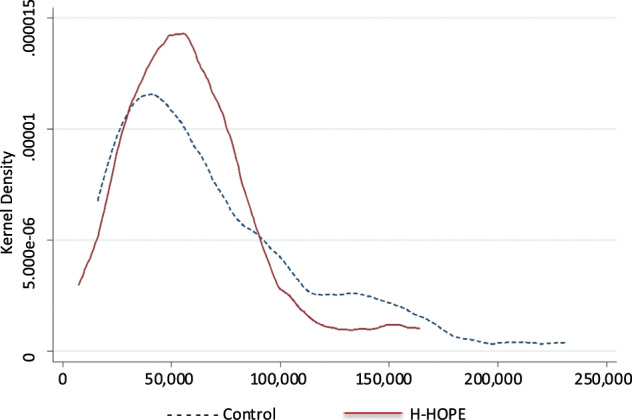
Distribution of hospital charges ($) by study group. The *y* axis represents the kernel density estimation (KDE). The *x* axis represents total hospital charges. Dotted line represents total hospital charges for the control group infants. Solid line represents total hospital charges for the H-HOPE intervention group infants.

Birth hospitalization charges by study condition are shown in Table 2. Compared to H-HOPE infants, total charges for the control infants were highly skewed toward higher charges. The mean cost for H-HOPE infants was $59,385 compared to $68,350 for the attention control group infants. Even though the mean of the control group was higher than the H-HOPE group, the median for the control group was lower than the H-HOPE group. The standard deviation, IQR (inter quarter range), and range of the control group were higher than the H-HOPE group. Figure 1 illustrates the differences in the distribution of hospital charges by study group using KDE. Compared to H-HOPE infants, total charges for the control infants were highly skewed toward the left with a long distributional tail showing higher charges. Examining the percentile-specific charges, the difference between the two study groups was larger at the higher the percentiles. At the 90th percentile the cost savings was $44,983 for the H-HOPE infants compared to $2,794 at the 10th percentile.

**Table 2:** Birth hospitalization charges distribution characteristic by study condition (N = 121).

Distribution Characteristic	H-HOPE (*n* = 60)	Control (*n* = 61)
Mean	$59,385	$68,350
Median	$56,604	$50,453
Standard deviation	$31,951	$45,971
Skewness	1.3575	1.2062
Minimum	$7,329	$16,063
Maximum	$164,145	$231,045
Percentile-specific		
10th percentile	$23,971	$26,765
25th percentile	$39,226	$35,391
75th percentile	$74,121	$86,264
90th percentile	$96,421	$141,404

**Fig. 2:**
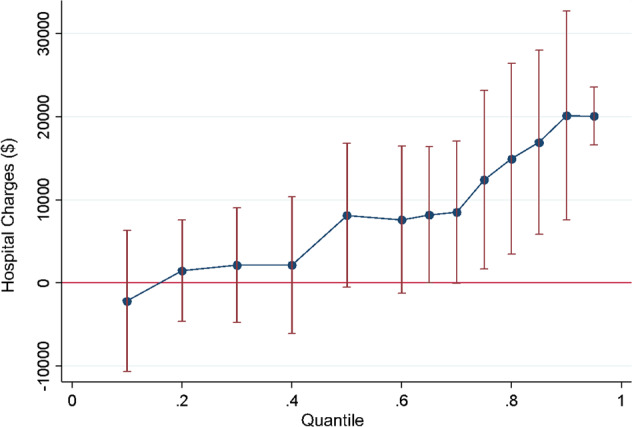
Estimated birth hospitalization savings ($) by quantile. This figure represents how the effect of H-HOPE varies over quantiles, and how the magnitude of the effect is stronger at higher quantiles. The *y* axis represents hospital charges. The *x* axis represents quantiles.

Program effects of H-HOPE using alternative estimation methods are summarized in Table 3. Across all three methods, H-HOPE resulted in a substantial cost savings. In the multiple linear regression analysis adjusting for infant and maternal characteristics, the mean charges for H-HOPE infants were $10,185, (*t* value = 2.56, *p* = 0.012) lower than control infants. Infants characteristics associated with higher charges included male sex, lower birth weight, and longer length of stay. Propensity score matching increases comparability of H-HOPE and control infants and showed the largest savings of $14,656. Using quantile regression, the estimated median difference of $8,154 for H-HOPE infants approached statistical difference (*p* = 0.065). Figure 2 shows the intervention effects on savings by quantile. There is a clear pattern of greater savings as the hospital charges increase. At the 75th percentile, the difference was $13,222 (p = 0.015).

**Table 3:** Effect of H-HOPE on infant birth hospitalization charges using alternative estimation methods (*N* = 121).

Estimation method	Regression coefficient	Standard error	*z*-test	*p* value	95% CI LL	95% CI HL
Multiple linear regression	−10,185	4,038	−2.52	0.013	−18,210	−2,161
Propensity score matching	−14,656	4,974	−2.95	0.003	−24,404	−4,907
Quantile regression						
Median	−8,154	4,363	−1.87	0.065	−16,824	516
75th percentile	−13,222	5,336	−2.48	0.015	−23,824	−2,620

*CI* confidence interval, *LL* lower limit, *HL* higher limit

Direct costs of the intervention training and implementation were $44,906.22 or a mean cost of $680.40 per infant in the H-HOPE group (Table 4). To obtain the average net cost difference per infant, we subtracted the mean in-hospital cost per infant from the mean charges of the birth hospitalization. Based on linear regression analysis showing the mean charges for H-HOPE infants were $10,185 lower (*p* = 0.013), after deducting the mean direct costs of the intervention per infant, the net savings per infant was $9,504.60.

**Table 4:** Direct Costs ($) of ATVV Intervention Training and Implementation (n = 66).

Cost description	Cost per Infant	Total Costs
Training costs		
Trainer Salary $50 per hour or $0.83 per minute for 720 min	9.09	600.00
Nurse-community advocate team		
Nurse Salary $34.98 including 21.25% fringes = $0.58 per minute for 720 min	6.36	419.77
Community advocate salary $17.48 including 21.25% fringes = $0.29 per minute for 720 min	3.18	209.81
In-hospital registered nurse		
Salary $28.85 per hour including 21.25% fringes = $0.48 per minute for 720 min	5.24	346.15
Materials Training manual; $0.05 per page for 10 pages for 4 guides for 2 nurses and 2 advocates	0.06	4.00
Training total	22.26	1,469.38
Implementation costs		
Nurse-community advocate team salaries—Two teaching sessions for an average of 1 h and 11 min per session		
Nurse	82.36	5435.76
Community advocate	41.18	2,717.88
In-hospital Nurse Salary for two ATVV sessions daily for 66 patients for an average of 23 days	533.60	35,217.60
Materials for Mother (*n* = 66) Handouts printed after downloading from pathways.org	1.00	66.00
Implementation Total	658.14	43,437.24
Total costs	680.40	44,906.62

## Discussion

In this study, preterm infants in the H-HOPE intervention group had substantially lower birth hospitalization charges. To provide a robust set of estimates we used three analytic approaches that controlled for maternal and infant characteristics. The H-HOPE intervention had consistently lower charges across all three statistical approaches: linear regression, propensity matching and quantile regression. The quantile regression clearly demonstrated that the cost savings was greatest among infants with the highest charges. In other words, H-HOPE likely had the greatest impact on the infants who had the highest resource use. We then calculated intervention costs to determine the net savings. The net savings far exceeded the costs of the intervention. Our findings are consistent with an earlier study that examined NICU charges and savings of an intervention for parents of premature infants [26]. Although our savings were substantially greater, this is likely a function of increasing healthcare costs and advances in preterm infant care over time. In addition, the H-HOPE intervention costs are substantially lower than the costs of a widely recognized developmental and early intervention model, Newborn Individualized Developmental Care and Assessment Program (NIDCAP) for healthcare professionals. However, NIDCAP includes a range of interventions such as modifications of the physical environment, coordination of the timing and organization of care, as well as, training healthcare professionals. NIDCAP costs to implement are also higher because in addition to training healthcare professionals, two full-time positions are recommended to support a 40 to 50-bed NICU further driving the costs higher to implement NIDCAP than H-HOPE [46].

The cost estimates of H-HOPE were conservative. We assumed the staff nurse completed the intervention twice daily. However, when mothers visited they provided the intervention with minimal assistance from the staff nurse. Assistance would typically include helping the mother move the infant in and out of the incubator (or open crib). This assistance would likely require less than the 20 minutes we used in our calculation. Also, staff nurses were not expected to offer H-HOPE on the weekend except to assist parents when visiting. Additionally, H-HOPE resulted in lower post-discharge healthcare utilization which represents additional cost savings not captured in this analysis [30].

A limitation of this study is the reliance on charge data rather than actual costs. While we requested actual costs and cost-to-charge ratios, hospital sites were not able to provide that information. There were also differences in how each site provided charge data, but each site provided the total charges. In addition, charge data were  not available for some infants.

This study was not designed to examine the mechanisms of H-HOPE that contribute to lower birth hospitalization costs. Cost savings were not expected to be related to length of stay because length of stay did not differ between study groups [13]. A likely explanation for lower charges is the significant impact of H-HOPE on behavioral, social interactive capabilities, feeding, and sucking organization [13, 29, 47, 48]. These outcomes directly contributed to greater physiologic stability and more rapid growth [13] which led to lower resource utilization and NICU charges.

### Implications for practice, research and policy

Early behavioral intervention for preterm infants has well documented benefits and has been recommended as the standard of care [28, 49, 50]. Yet, few NICUs provide this intervention outside of research. There is also strong evidence that parents need more in-hospital education and support regarding the unique needs of preterm infants. H-HOPE is an evidence based intervention that includes an infant-directed behavioral intervention and parent-directed participatory guidance regarding preterm infant’s unique needs and capacities. These strategies will lead to developmentally appropriate care and early social interaction that fosters growth and development of this highly vulnerable population. This cost analysis demonstrates that cost should not be a barrier to meeting preterm infant and parent needs. The costs of providing the in-hospital H-HOPE intervention were modest and the cost of the birth hospitalization was substantially lower. Research is now warranted to develop strategies to support widespread implementation. Examining the impact of H-HOPE beyond the initial hospitalization is also needed. Total savings of the H-HOPE intervention over the first year of life may be substantially greater than the savings documented in this analysis focused on birth hospitalization.
